# Supplementary material for: Effect of Green Light Replacing Some Red and Blue Light on Cucumis melo under Drought Stress
Source: Int J Mol Sci. 2024 Jul 10;25(14):7561. doi: 10.3390/ijms25147561 (PMC11276641; doi:10.3390/ijms25147561)

Table S1: Primers for real-time RT-PCR detection

Gene	Forward primer	Reverse primer
Action	5′-GTGATGGTGTGAGTCACACTGTTC-3′	5′-ACGACCAGCAAGGTCCAAAC-3′
MELO3C021064	5′-CCTTCAATGTCCTTCCATATCTCGCC-3′	5′-GGTTGTCGACCATCGGTAATTCGG-3′
MELO3C009386	5′-CAACAACTCTGTTTTAGCTCGCGCC-3′	5′-GAAGAAGGTGGGGACGGAACCTAATG-3′
MELO3C006862	5′-CAAAGTCCCTGCTCGTCTTCTTAGG-3′	5′-CCTTAGCGAGATGTTTGGAGGTCC-3′
MELO3C022435	5′-GTCGGTCCTTTGCTTTGTCCATGAG-3′	5′-CCGGTCAAAACCTTGATGTTTCCC-3′
MELO3C026609	5′-CGTTGCATCGAAGGAGGTCATGAAG-3′	5′-CATCGGAATGCCAAGACGAGTTGAC-3′
MELO3C014655	5′-GTAACCTCTTACGCCCAACTCACTG-3′	5′-CCTTGGACGAAACAATCGTGG-3′
MELO3C009329	5′-GACTATATCCAACGGCCGATCCTACC-3′	5′-CGAATGCCCCTTGAGAATTGTGC-3′
MELO3C025199	5′-CAGATGATAAGGTGGCTGGAC-3′	5′-CATGAGGAGCTGGTTTGACAG-3′
MELO3C019585	5′-CCTTCGTATGGCCATCCCATTACTC-3′	5′-GTTCTTCCAGTTGGCTGGGTTGTTG-3′

Table S2: Fundamental state of RNA-seq

Treatment	Raw Data Read	Valid Data Read	Q20 (%)	Q30 (%)	GC content (%)
CK_1	42129076	40487654	99.97	97.52	43
CK_2	42322200	40853436	99.97	97.45	43
CK_3	42219540	40678100	99.97	97.48	43
T3_1	41980568	40540574	99.97	97.57	43
T3_2	42643736	41026042	99.97	97.49	43
T3_3	42519908	40957042	99.97	97.55	43
CK_1	42129076	40487654	99.97	97.52	43

Figure S1: *RNA-Seq based classification of differential genes under different treatments in melon seedlings*

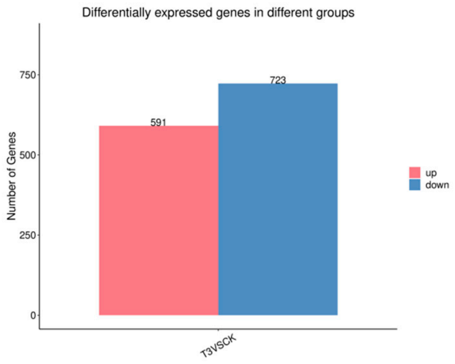

Figure S2: Transcriptome data qRT-PCR avalidation

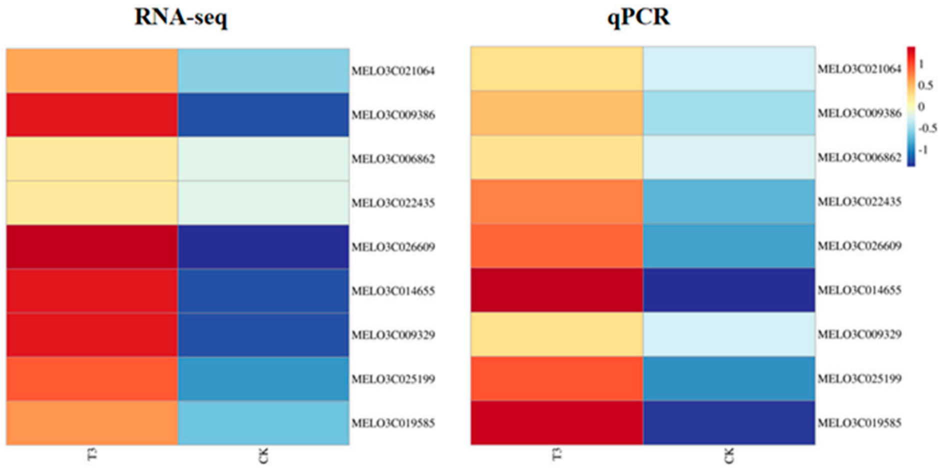

Figure S3: LC-MS based classification of differential metabolites under different treatments in melon seedlings

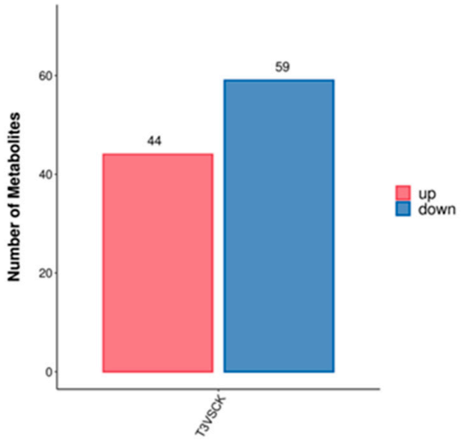

Supplement: Supplementary file 1 [file ijms-25-07561-s001.zip › ijms-3049713-supplementary.pdf]
